# IL-32 gamma reduces lung tumor development through upregulation of TIMP-3 overexpression and hypomethylation

**DOI:** 10.1038/s41419-018-0375-6

**Published:** 2018-02-21

**Authors:** Jaesuk Yun, Mi Hee Park, Dong Ju Son, Kyung Tak Nam, Dae Bong Moon, Jung Heun Ju, Ok Kyung Hwang, Jeong Soon Choi, Tae Hoon Kim, Young Suk Jung, Dae Yeon Hwang, Sang Bae Han, Do-Young Yoon, Jin Tae Hong

**Affiliations:** 1 College of Pharmacy and Medical Research Center, Chungbuk National University, Osongsaengmyeong1-ro 194-21, Heungduk-gu, Cheongju, Chungbuk 28160 Republic of Korea; 2 Department of Pharmacy, Wonkwang University, #460 Iksan-daero, Iksan-si, Jeonbuk 54538 Republic of Korea; 3 Department of Biomaterial Science, Pusan National University, Miryang, Kyungnam 50463 Republic of Korea; 4 Department of Bioscience and Biotechnology, Bio/Molecular Informatics Center, Konkuk University, Gwangjin-gu, Seoul 05029 Republic of Korea

## Abstract

The low expression of tissue inhibitor of metalloproteinase 3 (TIMP-3) is important in inflammatory responses. Therefore, inhibition of TIMP-3 may promote tumor development. Our study showed that expression of TIMP-3 was elevated in lL-32γ mice lung tissues. In this study, we investigated whether IL-32γ mice inhibited lung tumor development through overexpression of TIMP-3 and its methylation. To explore the possible underlying mechanism, lung cancer cells were transfected with IL-32γ cDNA plasmid. A marked increase in TIMP-3 expression was caused by promoter methylation. Mechanistic studies indicated that TIMP-3 overexpression reduced NF-κB activity, which led to cell growth inhibition in IL-32γ transfected lung cancer cells. We also showed that IL-32γ inhibits expression of DNA (cytosine-5-)-methyltransferase 1 (DNMT1). Moreover, IL-32γ inhibits the binding of DNMT1 to TIMP-3 promoter, but this effect was reversed by the treatment of DNA methyltransferase inhibitor (5-Aza-CdR) and NF-κB inhibitor (PS1145), suggesting that a marked increase in TIMP-3 expression was caused by inhibition of promoter hypermethylation via decreased DNMT1 expression through the NF-κB pathway. In an in vivo carcinogen induced lung tumor model, tumor growth was inhibited in IL-32γ overexpressed mice with elevated TIMP-3 expression and hypomethylation accompanied with reduced NF-κB activity. Moreover, in the lung cancer patient tissue, the expression of IL-32 and TIMP-3 was dramatically decreased at a grade-dependent manner compared to normal lung tissue. In summary, IL-32γ may increase TIMP-3 expression via hypomethylation through inactivation of NF-κB activity, and thereby reduce lung tumor growth.

## Introduction

Interleukin-32 (IL-32) was cloned as a gene induced by IL-18 and was formerly known as natural killer cell transcript 4^1^. IL-32 has six splice variants, IL-32α, IL-32β, IL-32γ, IL-32δ, IL-32ε, and IL-32ζ, which have been shown to have functional differences among these isoforms^1,2^. Since IL-32 modulates generation of pro and anti-inflammatory cytokines, such as tumor necrosis factor-alpha (TNF-α), IL-1β, IL-6, IL-10, and two C-X-C chemokine family members involved in inflammatory and/or autoimmune diseases^3–5^, there are different pathophysiological functions in the development of several diseases such as arthritis, psoriasis, ulcerative colitis, Crohn’s disease, chronic obstructive pulmonary disease and cancer that have been reported^3,6,7^. In our recent studies, IL-32 inhibited tumor growth in a xenograft animal model and carcinogen-induced colon tumor development^8^. However, the role of IL-32 on the carcinogen-induced-lung tumor growth, and action mechanisms have not been reported yet. Many cytokines are involved in cancer development in different ways^9^. IL-8 modulates endothelial cell proliferation and migration, thus promoting angiogenesis^10^. IL-6 increases antiapoptotic activity and, consequently, tumorigenic potency in basal cell carcinoma^11^. IL-10 inhibits tumor growth and metastasis in an animal model^12,13^, and it inhibits tumor metastasis through an NK cell-dependent tumor killing mechanism^14^. Moreover, a negative correlation between the expression of IL-10 and human colon cancer development has also been shown^15^. However, exact pathways in the cytokine-modulated tumor development are not clear.

The tissue inhibitor of metalloproteinase 3 (TIMP-3) gene, an insoluble 24-kDa glycoprotein that is produced by most cell types and is sequestered at the cell surface, is bound by components of the extracellular matrix^16^. TIMP-3 has been known to act as a tumor suppressor gene to inhibit tumor growth, invasion, and angiogenesis^17^. TIMP-3 is frequently found in meningiomas, glioblastomas, pancreatic endocrine carcinomas, and cervical or lung cancers^18,19^. Reduced TIMP-3 expression was associated with poor outcomes in lung cancer patients^20,21^. It has been reported that production of tumor necrosis factor α (TNFα) and IL-6 is elevated in TIMP-3 knockout mice tissues, and these activation and production subsequently leads to severe inflammation^22^. Mino et al. observed that TIMP-3 expression status is significantly correlated with pathologic stage and nodal involvement in resected non-small cell lung cancer (NSCLC)^21^. Munoz et al. also showed that after B16F10 tumor cell injection, more metastasized cells were found in the lungs of TIMP-3 knockout mice than in wild-type mice. A recent study indicated that IL-6 promoted lung cancer cell invasion and growth by loss of TIMP-3^16^. It has been reported that IL-27 suppressed tumor potential in prostate cancer by up-regulation of anti-angiogenesis-related genes including TIMP-3 and CXCL10 ^23^.

Changes of TIMP-3 expression by methylation have been significant in tumor growth, invasion and metastasis. Melanoma progression and cell migration were inhibited by TIMP-3 expression through its hypermethylation^24^. In glioblastoma development, TIMP-3 expression was reduced by methylation of the genes^18^. Post translational changes such as methylation and acetylation of genes resulted in the activation or inactivation of genes. Methylation of TIMP-3 could cause TIMP-3 inactivation, and its inactivation is associated with cancer development in the kidney, brain, breast, colon, esophagus, gastric, head and neck, and lung cancer^19,20^. However, the level of methylation of the TIMP-3 is variable among the cancer^25^. It was also reported that there is an association between TIMP-3 promoter methylation and better survival in lung cancer patients^26^. Several cytokines such as IL-1, IL-6 and TNF-β regulate TIMP-3 expression in variety tissues^27^. Moreover, cytokines changes methylation of gene for inactivation. Our microarray-wide analysis revealed that IL-32 mice showed significantly elevated levels of TIMP-3 in most tissues, especially lung, liver, kidney, endothelial cells and smooth muscle cells^28^. Thus, the exact roles and mechanisms of TIMP-3 and its methylation changed by IL-32 in lung tumor development were investigated in the present study.

## Results

### IL-32γ inhibits lung tumor growth and induces apoptosis

**Fig. 1:**
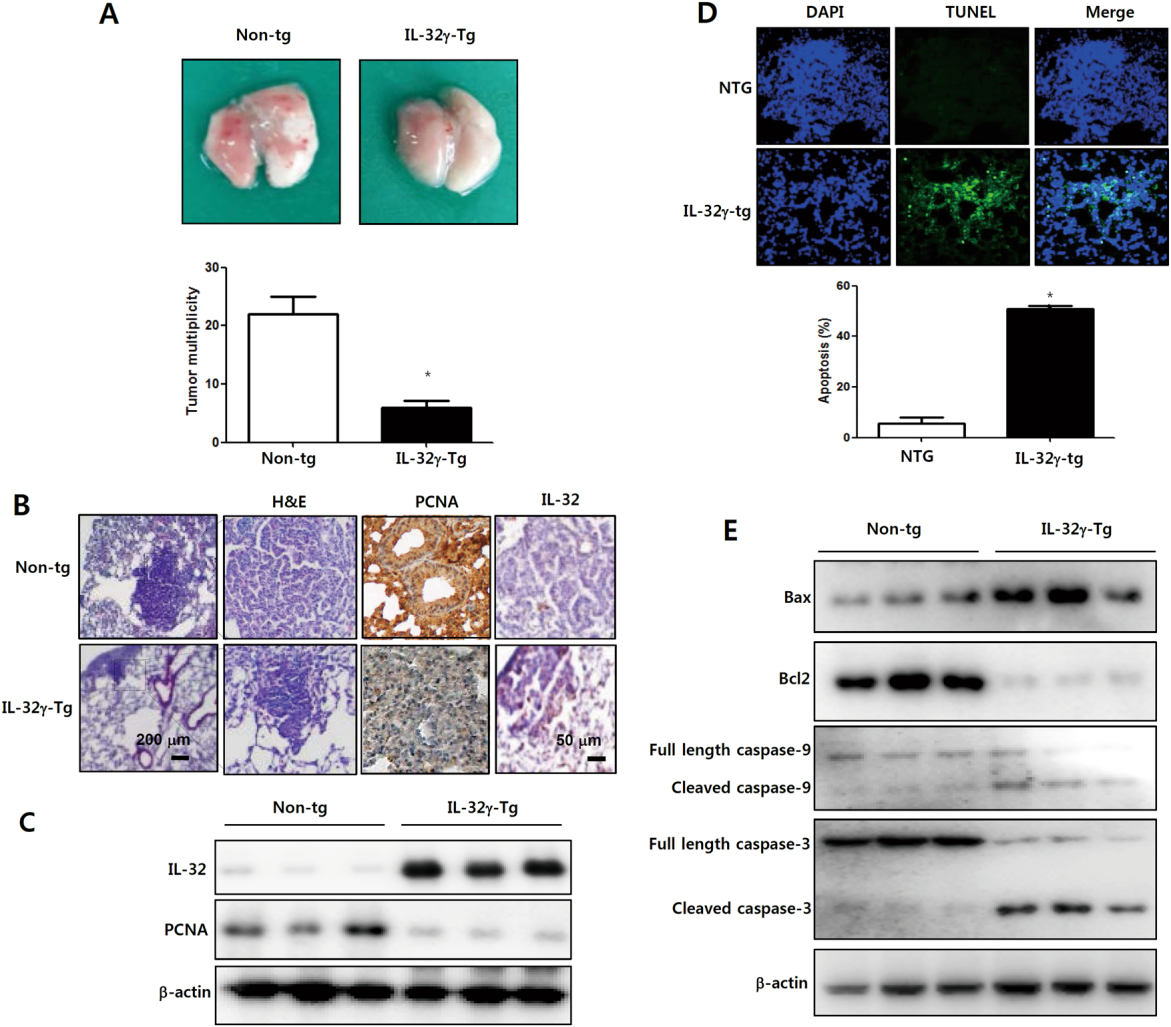
Effect of IL-32γ on lung tumor development. **a** Effect of IL-32γ on lung tumorigenesis in transgenic mice. The results are expressed as mean ± SD. *P < 0.05 compared with non-transgenic mice (*n* = 10). **b** Effect of IL-32γ on the expression of proliferation marker proteins and IL-32 and PCNA in tumor tissues determined by immunohistochemistry. **c** Effect of IL-32γ on the expression of IL-32 and PCNA in tumor tissues determined by Western blotting. **d** Effect of IL-32γ on apoptosis was determined by DAPI/TUNEL assay. Total number of cells in a given area was determined using a DAPI nuclear staining (fluorescent microscope). The green color in the fixed cells marks TUNEL-labeled cells. **e** Effect of IL-32γ on the expression of apoptotic proteins in tumor tissues determined by Western blotting with specific antibodies. β-actin protein was used as an internal control. Each band is representative of three independent experiments

Changes of cytokines in the lungs are critical factors for lung malignancies^29,30^. Several studies suggest that various cytokines are involved in lung tumor promotion or tumor suppression. However, the role of IL-32 on carcinogen-induced-lung tumor growth, and action mechanisms have not been reported yet. Therefore, we were interested in studying the effect of IL-32γ on lung cancer development. Lung tumorigenesis was induced using urethane injections. Thirty weeks after the initial urethane injections, the number of lung tumors in IL-32γ mice were significantly decreased (Fig. 1a). Tumor multiplicity was 5 ± 0.8 tumors per IL-32γ mice, but 20.9 ± 7.3 per wild type mice. The histological findings after hematoxylin and eosin staining indicated that the tumors in IL-32γ mice were significantly smaller and showed a few adenocarcinomas, however, tumors from wild type mice were well-differentiated lung adenomas (Fig. 1b). The proliferating cell nuclear antigen (PCNA) positive cells were smaller in IL-32γ mice than wild type mice (Fig. 1b). Western blotting data also showed that the protein level of PCNA was significantly decreased in IL-32γ mice compared to wild type mice (Fig. 1c). To determine whether apoptotic cell death contributed to the observed inhibitory effect of IL-32γ on lung tumor growth, we performed the TUNEL assay to detect apoptosis and Western blot to show the expression of apoptotic cell death regulatory proteins. We found that the percent of apoptotic cells was increased in IL-32γ mice compared to wild type mice (Fig. 1d). We also found that the expression of pro-apoptotic proteins, including Bax, cleaved caspase-3 and caspase-9, were increased but the expression of the anti-apoptotic protein, bcl-2, was decreased in the tumors of IL-32γ mice (Fig. 1e).

### IL-32γ inhibits cell growth and induces apoptosis in lung cancer cell lines

**Fig. 2:**
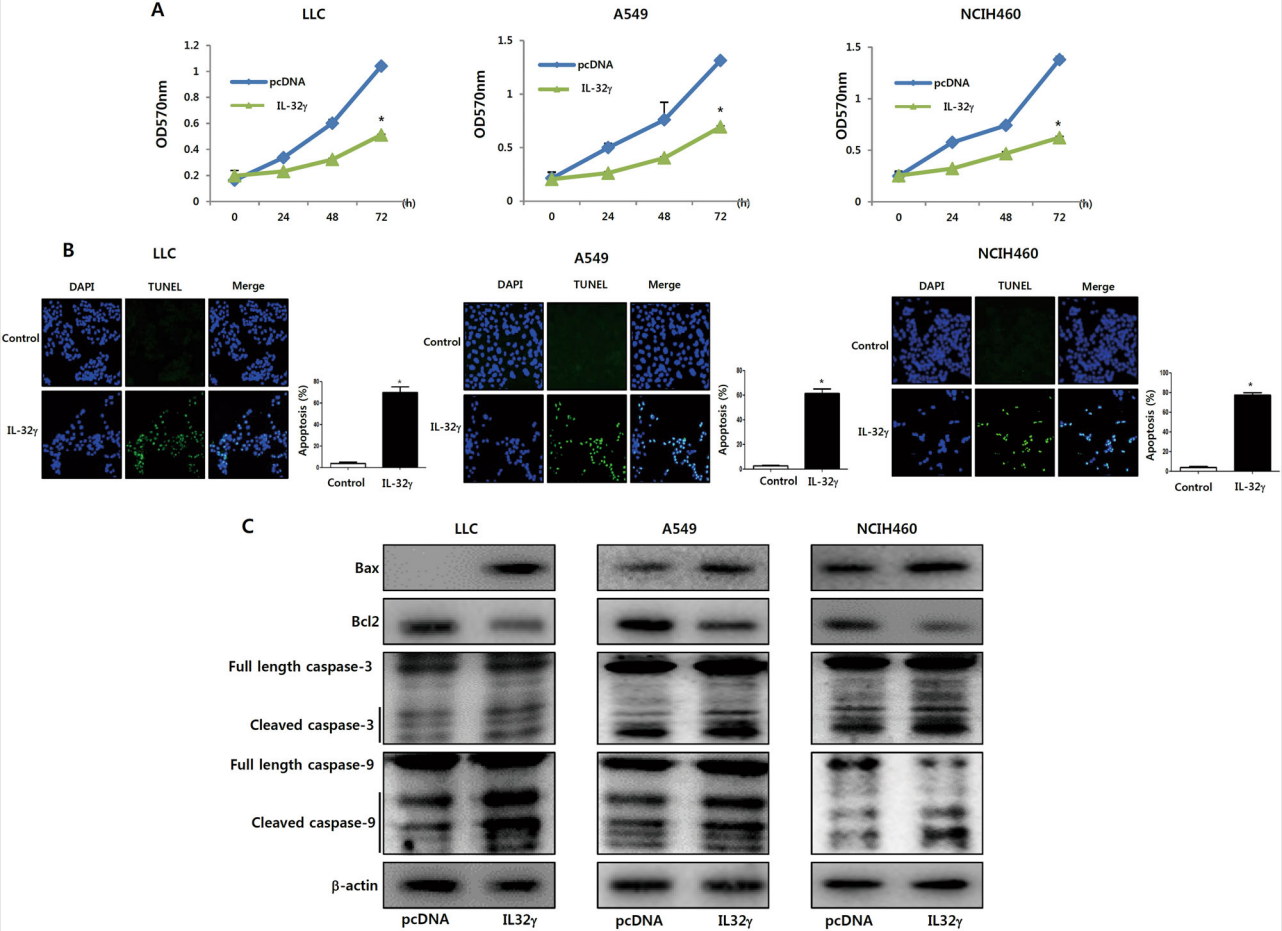
Effect of IL-32γ on cell viability and apoptosis in lung cancer cell lines. **a** Effect of IL-32γ on growth rates and apoptotic cell death in IL-32γ-transfected lung cancer cells. Lung cancer cells (1 × 10^4^) were transfected with pcDNA or IL-32γ plasmid for 24, 48 or 72 h and cell proliferation was determined by MTT assay. The results are expressed as mean ± s.d. of three experiments with each experiment performed in triplicate. *P < 0.05 compared with the vector-transfected lung cancer cells. **b** Effects of IL-32γ on apoptotic cell death. Lung cancer cell lines were transfected with IL-32γ and then labeled with DAPI and TUNEL solutions. Total number of cells in a given area was determined using a DAPI nuclear staining (fluorescent microscope). The green color in the fixed cells marks TUNEL-labeled cells. **c** Lung cancer cells were transfected with the vector or the IL-32γ for 24 h. Cell extracts were analyzed by western blotting. Each band is representative of three independent experiments

To further investigate whether the introduction of IL-32γ into cancer cells changes their growth in vitro, we carried out cell viability assay in lung cancer cells transfected with or without the IL-32γ gene. The introduction of IL-32γ resulted in the inhibition of lung cancer cell growth in a time-dependent manner, as compared with the cells transfected with an empty vector (Fig. 2a). To determine whether apoptotic cell death contributed to the observed inhibitory effect of IL-32γ on lung cancer cell growth, the apoptotic cell death was investigated. In the IL-32γ-overexpressing lung cancer cells, the percent of apoptotic cells was increased (Fig. 2b) and the expression levels of pro-apoptotic proteins Bax, cleaved caspase-3 and caspase-9 were increased. However, the expression levels of the antiapoptotic protein, bcl-2, was decreased in the lung cancer cells by IL-32γ (Fig. 2c).

### IL-32γ overexpression enhanced TIMP-3 expression via its hypomethylation

**Fig. 3:**
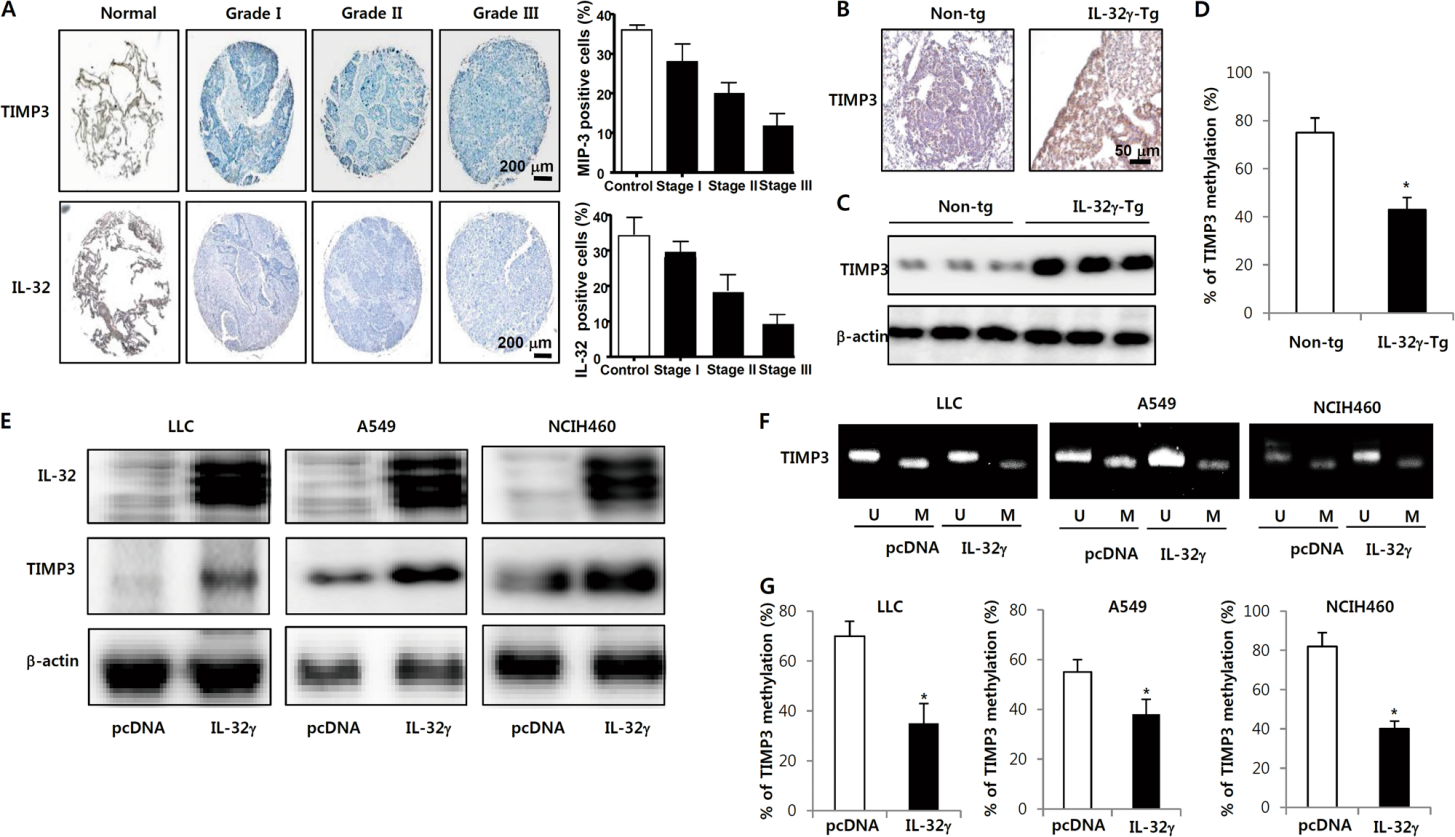
Effect of IL-32γ on the expression and promoter methylation of TIMP-3 in lung tumors and lung cancer cell lines **a** Expression of TIMP-3 and IL-32 is lower in human lung cancer tissues (*n* = 4) compared with normal lung tissues. Immunohistochemical staining was performed on a lung cancer tissue array using a TIMP-3 or IL-32 antibodies. The figure representative of 4 tissue samples. **b** Effect of IL-32γ on the expression of TIMP-3 in tumor tissues determined by immunohistochemistry. **c** Effect of IL-32γ on the expression of TIMP-3 in tumor tissues determined by Western blotting. **d** Effect of IL-32γ on the methylation of TIMP-3 in tumor tissues determined by qPCR. Scale bar: 50 μm or 200 μm. Each band is representative of three independent experiments. **e** Lung cancer cells were transfected with pcDNA or IL-32γ for 24 h. Cell extracts were examined for expression of IL-32 and TIMP-3 by Western blotting. **f**,** g** TIMP-3 promoter methylation levels were determined by methylation-specific PCR (**f**) or quantitative methylation-specific PCR by qPCR (**g**) in cell extracts from pcDNA or IL-32γ transfected lung cancer cells

Growing evidences have demonstrated that expression of TIMP-3 is significant in the development of tumor and its expression is reduced in lung tumor patients^31^. We found that the expression of TIMP-3 was decreased in patient-derived lung tumor tissues in a grade-dependent manner (Fig. 3a). Accompanied with TIMP-3 expression, the IL-32 expression was also decreased in patient-derived lung tumor tissues in a grade-dependent manner (Fig. 3a). We also found that the expression of TIMP-3 was dramatically increased in brain, kidney and lung tissue, and IL-32 was highly expressed in kidney and lung tissue in IL-32γ mice (Supplementary Fig.1). Immunohistochemistry analysis showed that higher TIMP-3 protein expression was more common in tumors of IL-32γ mice than in wild type mice (Fig. 3b). Agreeing with IHC data, the protein level of TIMP-3 was increased in the tumor tissues of the IL-32γ mice than in the wild type mice by Western blotting (Fig. 3c). To verify whether TIMP-3 expression could be mediated by promoter methylation, quantitative methylation-specific PCR were used to determine TIMP-3 promoter methylation. We showed that methylation of TIMP-3 was decreased in the tumor tissues of the IL-32γ mice than in the wild type mice by qPCR (Fig. 3d). To further investigate whether the increase of TIMP-3 expression has an inhibitory effect on lung cancer cell growth through decreasing methylation of TIMP-3, IL-32γ transfected lung cancer, we determined the expression of TIMP-3 and its methylation in lung cancer. We found that significant increase of TIMP3 expression in the IL-32γ transfected lung cancer cell compared to that in mock transfected lung cancer cells. In accordance with patient tissue and animal tissue, TIMP-3 expression was increased by the introduction of IL-32γ plasmid in LLC, A549 and NCIH460 lung cancer cell lines (Fig.3e). We further investigated how the expression of TIMP-3 is elevated by IL-32γ. Change in methylation of TIMP-3 could be inversely associated with TIMP-3 expression. First, we determined the methylated TIMP-3 and unmethylated TIMP-3 in lung cancer cell lines. We showed that methylated TIMP-3 was decreased, but unmethylated TIMP-3 was increased in the IL-32γ transfected group compared to the control group (Fig.3f). But, the TIMP-3 methylation was not significantly changed in other cancer cell lines including the colon, liver and skin cancer cell lines (Supplementary Fig. 2,A). Moreover, methylated TIMP-3 and unmethylated TIMP-3 were not changed in these cell lines (Supplementary Fig. 2,B). We also determined the effect of IL-32γ on the TIMP-3 methylation in lung cancer cell lines. IL-32γ resulted in a readily noticeable decrease in TIMP-3 methylation in the lung cancer cell lines transfected with IL-32γ (Fig. 3g). These data suggest that TIMP-3 methylation-dependent expression of TIMP-3 could be associated with the inhibitory effect of IL-32γ on lung cancer cell growth. We further identified the effect of other isoforms of IL-32, including IL-32α and IL-32β, on TIMP-3 promoter methylation in A549 and NCIH460 lung cancer cell lines. We showed that other isoforms of IL-32 had no significant effect on TIMP-3 promoter methylation (Supplementary Fig.3A). Moreover, the methylation of other genes including *E-cadherin*, cysteine dioxygenase 1 (*CDO-1*), *TERT* and *p16*, which methylation are important for lung cancer development, were not significantly changed (Supplementary Fig.3B). We further confirmed the methylation of TIMP-3 by IL-32γ using siRNA of IL-32γ. We found that knockdown of IL-32γ reversed IL-32γ decreased methylation of TIMP-3, but prevented the increasing effect of IL-32γ on the TIMP expression (Supplementary Fig.3C). From these results, we suggest that IL-32γ selectively affects TIMP-3 methylation in lung cancer, resulting in the inhibition of lung cancer cell growth.

### Decrease of NF-κB activity in tumor tissue of IL-32γ mice

**Fig. 4:**
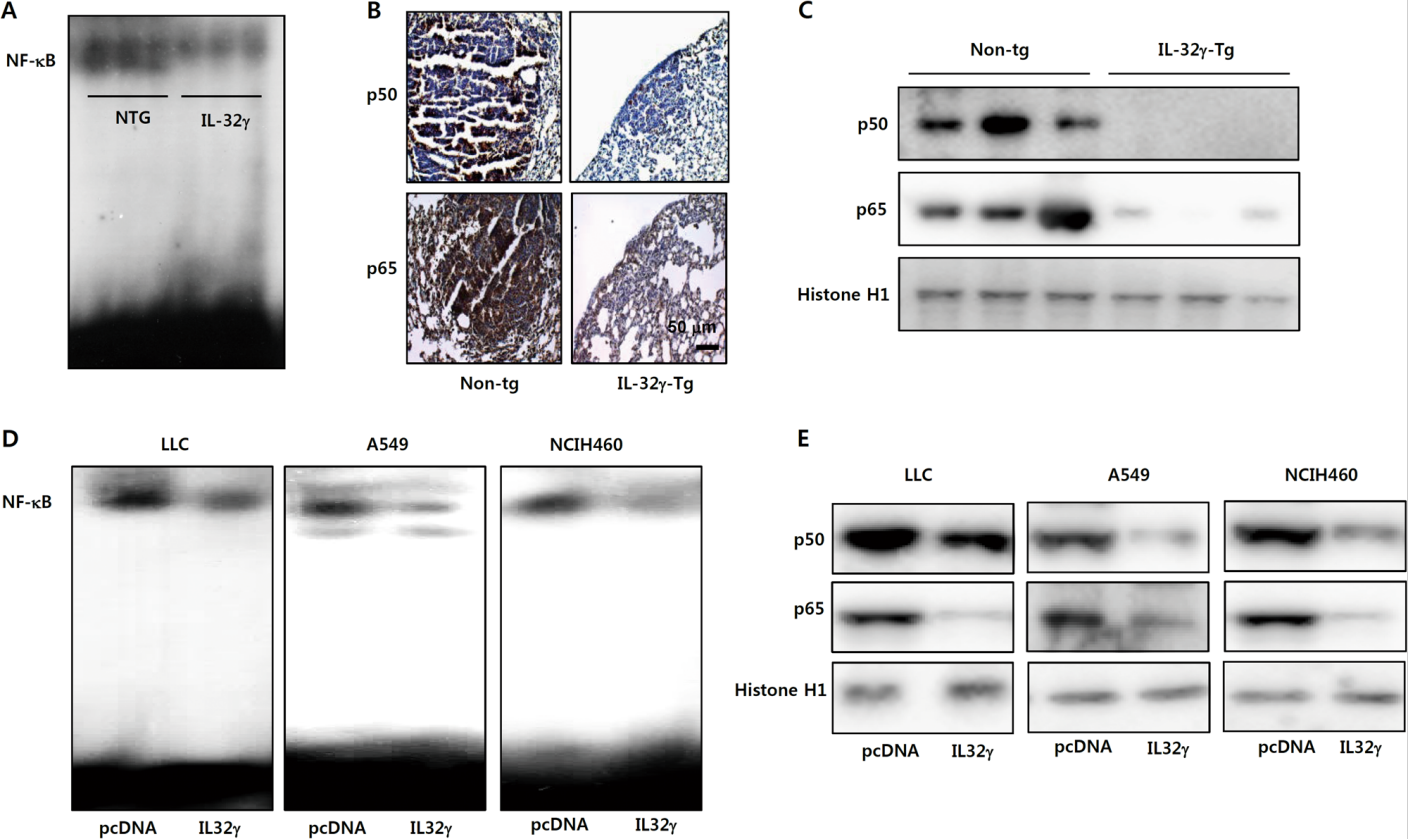
Effect of IL-32γ on NF-κB signaling and on the binding of DNMT1 in TIMP-3 promoter. Effect of IL-32γ on NF-κB activation in tumor tissues and lung cancer cells. **a** DNA-binding activity of NF-κB was determined by electromobility shift assay in the nuclear extracts of non-tg or IL-32γ-tg mice tumor samples. **b**, **c** Nuclear translocation of p50 and p65 of murine tumors was determined by immunohistochemistry (**b**) and Western blotting (**c**). Each image and band is representative of three independent experiments. **d** DNA-binding activity of NF-κB was investigated using electromobility shift assay in nuclear extracts of lung cancer cells that were transfected by IL-32γ. **e** Nuclear translocation of p50 and p65 was determined by Western blotting

It was found that IL-32γ could down-regulate Rel/p50 which is associated with TIMP-3 expression. Thus we determined whether NF-κB transcriptional factors should be activated according to the decrease of TIMP-3 expression. Moreover, NF-κB is critical in tumor growth. The lower DNA binding activity of NF-κB was found in the lung tissues of IL-32γ mice compared to that of wild type mice (Fig. 4a). In addition, the translocation of p50 and p65 was much less in the lung tumor tissue of IL-32γ mice compared to those in wild mice lung tissues by immunohistochemistry (Fig. 4b) and Western blotting (Fig. 4c). We also observed marked repression of DNA binding activity of NF-κB in lung cancer growth in lung cancer cells transfected with IL-32γ (Fig. 4d). Agreeing with in vivo data, the translocation of p50 and p65 was also reduced in the lung cancer cells employed with IL-32γ (Fig. 4e).

### IL-32γ methylates TIMP-3 promoter by NF-κB-dependent activation of DNMT1

**Fig. 5:**
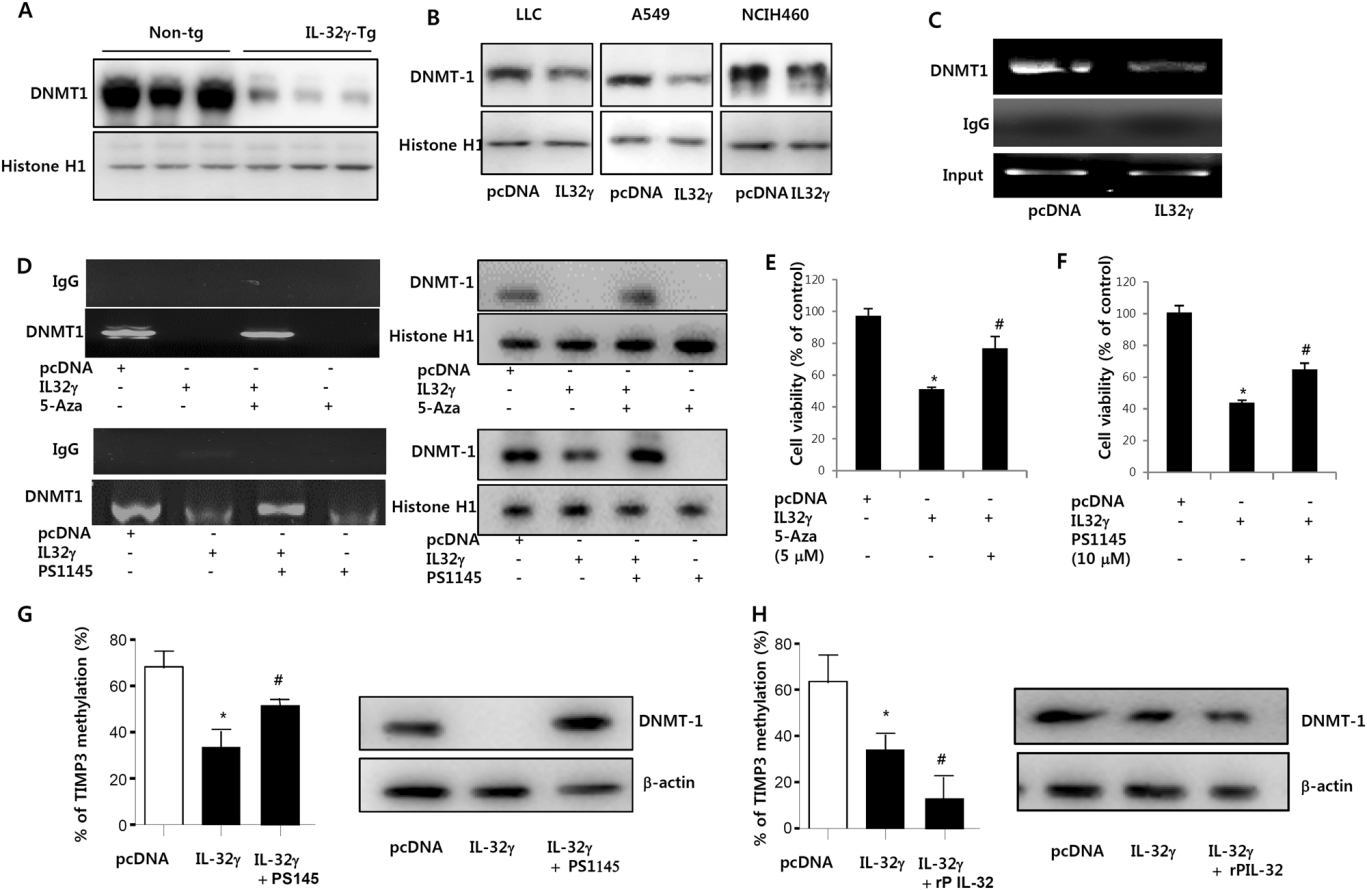
Effect of IL-32γ on the binding of DNMT1 in TIMP-3 promoter. **a** Expression of DNMT1 of murine tumors was determined by Western blotting. **b** Expression of DNMT1 in nuclear extracts of lung cancer cells transfected by IL-32γ was determined by Western blotting. **c** A549 lung cancer cells were transfected with pcDNA or IL-32γ plasmid for 24 h. Reduced binding of DNMT1 by IL-32γ in TIMP-3 promoter was determined by ChiP assay. **d** Effect of DNA methyltransferase inhibitor (5-Aza-CdR; 5 μM) on reduced binding of DNMT1 by IL-32γ in TIMP-3 promoter was determined by ChiP assay. Effect of NF-κB inhibitor (PS1145; 10 μM) on reduced binding of DNMT1 by IL-32γ in TIMP-3 promoter was determined by chip assay. TIMP-3 promoter was determined by ChiP assay. Each band is representative of three independent experiments. **e**,** f** Effect of DNA methyltransferase inhibitor (5-Aza-CdR; 5 μM) (**e**) or NF-κB inhibitor (PS1145; 10 μM) (**f**) on cell viability was determined by MTT assay. **g** Effect of NF-κB inhibitor (PS1145; 10 μM) on TIMP-3 methylation and Expression of DNMT1. **h** Effect of recombinant protein (rP) of p50 (50 ng/ml) on TIMP-3 methylation and Expression of DNMT1 in A549 cells. Allthe experiment were performed three times with duplicates. *P < 0.05 compared with the lung cancer cells transfected with vector. ^#^*P* < 0.05 compared with the lung cancer cells transfected with IL-32γ alone

It is noteworthy that several genes could be methylated through NF-κB-dependent DNMT1 activity. So, we investigated whether IL-32 inhibits NF-κB-dependent DNMT1 activity, thereby decreasing TIMP-3 promoter methylation. We found that DNMT1 expression was decreased in the lung tumor tissue of IL-32γ mice (Fig. 5a), and this effect was confirmed in the lung cancer cell lines (Fig. 5b). Next, we defined that DNMT1 directly binds with TIMP-3 promoter region by ChiP assay and IL-32γ inhibits the binding of DNMT1 to TIMP-3 promoter (Fig. 5c). But this effect was reversed with the treatment of 5 μM of DNA methyltransferase inhibitor (5-aza-2′-deoxycytidine; 5-Aza-CdR) (Fig. 5d). Moreover, DNA binding activity of DNMT1 onto the TIMP-3 promoter region was reversed by NF-κB inhibitor PS1145 (Fig. 5d). We also showed that the IL-32γ-dependent cell growth inhibitory effect was reversed by DNA methyltransferase inhibitor (Fig. 5e) and NF-κB inhibitor (Fig. 5f). In addition the IL-32γ decreased TIMP methylation was reversed by the treatment with NF-κB inhibitor in A549 cells (Fig. 5g). Moreover, we also investigated the effect of NF-κB activation on TIMP-3 methylation and expression of DNMT expression in the lung cancer cells (A549 cells) treated with recombinant protein p50. We found that TIMP methylation was further decreased in the p50 recombinant protein treated cells, and the DNMT1 expression was also further lowered (Fig. 5h). These results clearly indicate that IL-32γ in lung cancer cells may promote TIMP-3 promoter methylation via increased DNA binding activity of DNMT1 on TIMP-3 promoter in a NF-κB-dependent manner.

### Involvement of IL-32γ on the metastasis signaling through regulation of TIMP-3

**Fig. 6:**
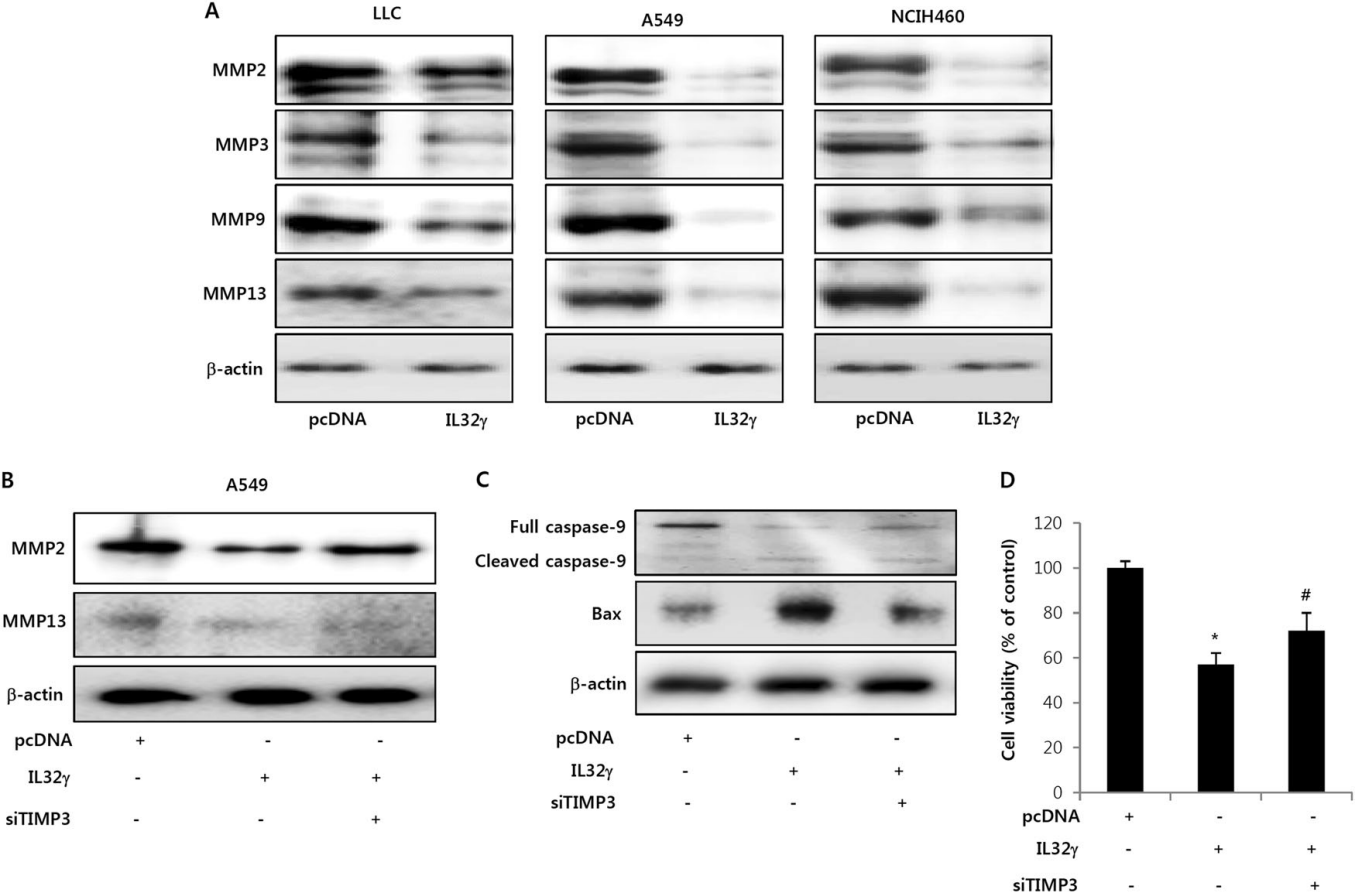
Effect of IL-32γ on the expression of metastasis related proteins. **a** Lung cancer cells were transfected with pcDNA or IL-32γ plasmid for 48 h. Cell lysates were analyzed by Western blotting for detection of MMP2, MMP3, MMP9 and MMP13 expression. β-actin protein was used as an internal control. Each band is representative for three experiments. **b-d** Lung cancer cells were co-transfected with the IL-32γ and TIMP-3 siRNA for up to 48 h. Effect of siTIMP-3 on the expression of metastatic proteins (**b**) and apoptotic protein (**c**) and was determined by Western blotting. Cell growth was measured by MTT assay (**d**). The results are expressed as mean ± s.d. of three experiments with triplicate tests in each experiment. *P < 0.05 compared with the lung cancer cells transfected with vector. ^#^*P* < 0.05 compared with the lung cancer cells transfected with IL-32γ alone. The values are average percentages of vector control over three independent experiments

It has been reported that most NSCLC patients are present with locally advanced or metastatic diseases^32^. Tumor malignancies are correlated with advanced tumor progression and aggressive metastasis^32^. A recent study demonstrated that higher TIMP-3 expression was associated in tumor development by regulation of invasion and metastasis^33^. Another research group also suggested that lung cancer cell invasion is promoted by the loss of TIMP-3^16^. It has been reported that tumor growth was inhibited by up-regulation of anti-angiogenesis-related genes^23^. So, we wondered if TIMP-3 regulated by IL-32γ finally reduces the expression of metastasis related proteins such as MMP-2, MMP-3, MMP-9 and MMP-13. We found that the expression of these proteins was dramatically reduced by the introduction of IL-32γ (Fig. 6a). However, the MMP expression was reversed by knockdown of TIMP3 (Fig. 6b). Moreover, increased cleaved caspases and bax expression were reversed by introduction of siTIMP-3 (Fig. 6c), resulting in the reversed effect on cell viability (Fig. 6d). These data suggest that TIMP-3 methylation-dependent expression of TIMP-3 could be associated with the inhibitory effect of IL-32γ on lung cancer cell growth.

## Discussion

In this study, we found that IL-32γ mice have an inhibitory effect on carcinogen-induced lung tumor development. This observation extended our previous studies showing anti-tumor effects of IL-32 in melanoma, colon and prostate tumor^8^. We also found that the expression of TIMP-3 was elevated in IL-32γ mice lung tumor tissues. Several other studies have demonstrated that changes of TIMP-3 expression have been significant in tumor growth, invasion and metastasis^17^. Melanoma progression and cell migration were inhibited by TIMP-3 expression^34^. In glioblastoma development, TIMP-3 expression was reduced^18^. TIMP-3 hypermethylation was found in HPV-positive oropharyngeal squamous cell carcinoma as well as head and neck squamous cell carcinoma incidence^35^. Several studies also demonstrated that TIMP-3 is associated with lung tumor development in patients. In lung cancer patients, the reduced TIMP-3 expression was associated with poor outcomes, and the overall survival rate was lower^16^. B16F10 tumor metastasis was higher in lungs of TIMP-3 knockout mice^24^. Moreover, TIMP-3 suppresses tumor angiogenesis in MMP-2-downregulated lung cancer^36^. Agreeing with these data, our data also demonstrated that upregulation of TIMP-3 inhibits lung tumor development by IL-32γ.

It has been reported that higher TIMP-3 expression, but lower promoter methylation, was associated in lung tumor developement^16,20,21^. To our knowledge, this is the first study to reveal that expression and methylation of TIMP-3 are positively correlated with IL-32-induced lung tumor inhibition. Moreover, several other studies have also demonstrated that methylation of genes by cytokines are closely related to tumor growth. IFN-γ also regulated TRIM16 gene expression and methylation in melanoma metastasis^37^. IL-10 also reduced wnt gene methylation, and thereby inhibits colon cancer incidence^38^. It is also observed that IL-6 expression increased TIMP-3 methylation in lung adenocarcinoma and also increased cyp enzymes methylation in colon cancer^39^. IL-23 expression via its methylation resulted in the promotion of metastasis of colorectal cancer^40^. We, in the present study, found that IL-32γ induced hypomethylation of TIMP-3 in lung cancer cell lines. In addition, we also found that knockdown of IL-32γ reversed IL-32γ decreased methylation of TIMP3, but prevented the increasing effect of IL-32γ on TIMP expression. But, we found that IL-32α and IL-32β were not significantly induced by hypermethylation of TIMP-3. The hypomethylation effects of IL-32γ could be selective only on TIMP-3 since we found that IL-32 did not significantly stimulate hypomethylation of cadherin, CDO-1 and TERT. In the tumor tissue of IL-32γ overexpressed transgenic mice, the methylation of TIMP-3 was inhibited. Moreover, the TIMP-3 methylation pattern was reversely associated with lung tumor stage of patients, but the TIMP-3 expression was closely related with tumor development. These data indicated that IL-32γ could change methylation patterns of TIMP-3, increased TIMP-3 expression resulting in the tumor growth inhibition.

It is unclear how IL-32γ induces hypomethylation of TIMP-3. However, it is noteworthy that several genes could be methylated through NF-κB-dependent DNMT1 activity. Phosphorylation of RELA/p65 promotes DNMT-1 to represses BRMS1 tumor metastasis^41^. It was also found that p16 gene promoter methylation was significantly inhibited by knockdown of NF-κB^42^. CDX1 methylation was also increased by NF-κB activating intestinal metaplasia^43^. Methylation of O6-methylguanine DNMT was also increased by NF-κB-dependent activation of DNMT^44^. Cytokines-induced gene methylation was also dependent on NF-κB activation. IL-1β methlylated cadherin through inhibition of NF-κB in H. pylori induced gastric cancer incidence^45^. It has been reported that IL-1β induces methylation of NF-κB by upregulation of DNMT in benign meningioma^46^. CDX1 promoter methylation was highly consistent with NF-κB signal activation, but inversely associated with TNF-α expression in the carcinogen-induced stomach cancer development^43^. TNF-α-induced hypermethylation of PRKCDBP was associated with NF-κB activation in the tumor suppressive effect of PRKCDBP in colorectal cancer^47^. TNF-β stimulation causes cadherin and collagen 1/g1 methylation by DNMT activation in the EMT and metastasis in ovarian cancer^48^. It was also important to note that IL-1β stimulated H. pylori-induced gastric inflammation and cadherin methylation via DNMT activation in IL-1ra knockout mice^49^. We showed that the expression of DNMT1 was decreased in IL-32γ transgenic mice accompanied decreased translocation of p50 and p65. In addition, the binding of DNMT1 in TIMP-3 promoter was decreased by the introduction of IL-32γ. We also found that decreased binding of DNMT1 was reversed by treatment of 5-Aza, inhibitor of methyltransferase and NF-κB inhibitor. Moreover, we found that TIMP methylation was further decreased in the p50 recombinant protein treated cells, and the DNMT1 expression was also further decreased. These data indicate that IL-32γ inhibited binding of DNMT1 to TIMP-3 promoter by decreasing the activity of NF-κB. Taken together, TIMP-3 overexpression by hypomethylation on its promoter by IL-32γ may play an important role in lung tumor development. In fact, DNMT has a NF-κB binding site in the promoter region. Even though many researchers have demonstrated that IL-32 could activate NF-κB^50,51]^, we previously found that IL-32 inactivates NF-κB in several cancer cells^8^. These observations suggest that IL-32γ could cause hypermethylation of TIMP-3 leading to an increase of expression which could act as a tumor suppressive gene. Conclusively, our present data indicate that IL-32γ inhibits methylation of TIMP-3, and thus increased TIMP-3 expression in lung tumor resulted in inhibition of lung tumor development.

## Methods

### Cell culture

NCIH460 and A549 human lung cancer cells as well as other cells such as LLC, SW480, HepG2, Sk-Mel-28 were obtained from the American Type Culture Collection (Manassas, VA, USA). RPMI1640, penicillin, streptomycin, and fetal bovine serum were purchased from Invitrogen (Carlsbad, CA, USA). Cancer cells were grown in RPMI1640 with 10% fetal bovine serum, 100 U/ml penicillin, and 100 μg/ml streptomycin at 37 °C in 5% CO_2_ humidified air.

### Cell viability assay

For MTT assay, 10% vol/vol of 5 mg/ml 3-(4,5-dimethylthiazol-2-yl)-2,5-diphenyltetrazolium bromide (MTT; Sigma) diluted in phospahte-buffered saline (PBS) was added to A549 and NCIH460 cell cultures. After 2 h of incubation, the medium was aspirated, and DMSO was added. Absorbance was measured at 570 nm. The data were normalized to their respective controls and are presented as a bar graph.

### DNA isolation, bisulfite conversion and methylation specific PCR

Genomic DNA was isolated using the Exgene^TM^ Tissue SV mine (GeneAll), 250 ng DNA was bisulfite modified using the EpiTect Bisulfite kit (Qiagen) and eluted in 20 μl elution buffer and 50 ng of converted DNA was amplified by PCR using Taq DNA Polymerase (WizPure). Primer sequences specific for unmethylated DNA were 5′-TTTTGTTTTGTTATTTTTTGTTTTTGGTTTT-3′ (sense) and 5′-CCCCCAAAAACCCCACCTCA-3′ (antisense) (122 bp), and methylated DNA were 5′-CGTTTCGTTATTTTTTGTTTTCGGTTTC-3′ (sense) and 5′-CCGAAAACCCCGCCTCG-3′ (antisense) (116 bp). PCR reactions were performed in 20 μL reactions with an annealing temperature of 60 °C, and products were visualized on 1% agarose gels.

### Real-time quantitative methylation-specific PCR

Sodium bisulfite-treated genomic DNA was amplified using fluorescence-based real-time methylation-specific PCR using SYBR Green qPCR MasterMix (Life Technologies, Carlsbad, CA). The methylation status of the TIMP-3 gene was examined using actin as the internal control for DNA quantification. Actin or GAPDH contains no CpG dinucleotides and is not affected by DNA methylation status or sodium bisulfite treatment. The following primers were used: human methylated (TIMP-3) forward primer, 5′-TCGGGTTGTAGTAGTTTCGTC-3′ and human methylated (TIMP-3) reverse primer, 5′-ACGATAAACCCGAACCAA-3′, and actin-forward, 5′-TGGTGATGGAGGAGGTTTAGTAAGT-3′ and actin-reverse, 5′-AACCAATAAAACCTACTCCTCCCTTAA-3′. Methylated E-cadherin forward primer was 5′-TTAGGTTAGAGGGTTATCGCGT-3′ and E-reverse primer was 5′-TAACTAAAAATTCACCTACCGAC -3′, methylated cysteine dioxygenase1 (CDO1) forward primer was 5′-CGAATTATAGCGGCGGAGGT-3′ and reverse primer was 5′-AAATCGCGTAAACTCCGCG-3′, and methylated TERT forward primer was 5′- GAGTTTGGATTTTTGGGAAGTTT-3′ and reverse primer was 5′- TAAAACCAACATCTAATCACATCCC -3′. Mouse methylated (TIMP-3) forward primer was GAGAGGCGGTGGGCGTAG and mouse methylated (TIMP-3) reverse primer was CGAAAATATAAACTAAACGCGTCCT, and GAPDH-forward, 5′-AATAGTTATTTTAAGTATTTATGAAATAAG-3′ and GAPDH-reverse, 5′-TAACTACCTCAACACCTCAAC-3′. The bisulfite-treated *in vitro* methylated DNA (SssI methyltransferase, New England Biolabs) was used as a positive control. Each reaction was performed in triplicate. The bisulfite-treated *in vitro* methylated DNA was included in each run to serve as the 100% methylated reference for calculating the relative methylation percentages of DNA samples based on the relative 2^(−ΔΔ^*CT*^)^ quantitation approach. Samples were considered to show positive methylation when the percentage of methylation was more than 50%, whereas a finding of less than 50% was considered as negative. And then we analyzed the methylated TIMP3 in control and IL-32γ transfected group using qPCR.

### Cytokine assay

Liver tissues were homogenized with protein extraction solution (PROPREP, iNtRONBiotechnology, Korea) and measured quantity of IL-6, IL-1β, and TNF-α in total proteins (1 mg) using mouse assay kit (R&D systems, Minneapolis).

### Animals

The IL-32γ mice were obtained as described elsewhere^8^. The IL-32γ overexpressed mice was generated by injection of IL-32 γ into B6D2F1 mice, and these founder mice were back-crossed into the C57BL6/J background mice for eight generations. After confirming the gene transmitted, the mice were housed and bred under specific pathogen free conditions at the Laboratory Animal Research Center of Chungbuk National University, Korea (CBNUA-929-16-01). The mice (*n* = 15) were maintained in a room with a constant temperature of 22 ± 1 °C, relative humidity of 55 ± 10%, and 12-h light/dark cycle, and fed standard rodent chow (Samyang Co., Gapyeong, Korea) and purified tap water ad libitum.

### Carcinogenesis protocols

Eight-week-old IL-32γ mice were used. Tumors were induced by a single i.p. injection of 1 mg/g urethane (ethyl carbamate; Sigma-Aldrich, St. Louis, MO) once a week for 10 weeks. Mice were euthanized at time points up to 6 months after injection of carcinogen. At the time of sacrifice, lungs were lavaged, perfused, and fixed in ice-cold Bouin’s fixative solution (Sigma-Aldrich) for 24 h. After fixation, lungs were used for surface tumor number and diameter measurements, and embedded in paraffin. Tumors on the lung surface were enumerated by at least two experienced readers, blinded to sample identifiers under a dissecting microscope; tumor counts were averaged and statistically analyzed. Tumor diameters were measured using Fisherbrand Traceable digital calipers (Fisher Scientific, Asheville, NC).

### Human samples

Human lung cancer tissue array (LC1502) containing lung tumors from patients and samples of normal tissue were purchased from US Biomax (Rockville, MD, USA). Tissue arrays were subjected to immunohistochemistry analysis.

### Immunohistochemistry

Immunohistochemistry was done as described previously^8^.

### Western blot analysis

Western blot analysis was done as described previously^50^. Antibodies were obtained from several providers. Cleaved caspase-3 (9661), Cleaved caspase-9 (7237), Caspase-3 (9662), Caspase-9 (9508) and p65 (8242) were from Cell Signaling (Danvers, MA, USA), β-actin (sc-130300), PCNA (sc-25280), Bax (sc-7480), p50 (sc-114), MMP-2 (sc-13595), MMP-3 (sc-6839), MMP-13 (sc-12363), normal mouse IgG (sc-2025) and MMP-9 (sc-127590 were purchased from Santacruz (Dallas, TX, USA) and IL-32 (513601) was obtained from BioLegend, (San Diego, CA USA). TIMP3 (ab39184) and DNMT1 (ab19905) were obtained from Abcam (Cambridge, MA, USA).

### Electromobility shift assay

Briefly, 1 × 10^6^ cells/ml were washed twice with 1× PBS, followed by the addition of 1 ml of PBS, and then cells were scraped into a cold Eppendorf tube. Cells were spun down at 15,000 g for 1 min, and the resulting supernatant was removed. Solution A (50 mM HEPES, pH 7.4, 10 mM KCl, 1 mM EDTA, 1 mM EGTA, 1 mM dithiothreitol, 0.1 μg/ml phenylmethylsulfonyl fluoride, 1 μg/ml pepstatin A, 1 μg/ml leupeptin, 10 μg/ml soybean trypsin inhibitor, 10 μg/ml aprotinin, and 0.5% Nonidet P-40) was added to the pellet in a 2:1 ratio (v/v) and incubated on ice for 10 min. 0.5 g of tumor tissue was chopped into 1.5 ml of solution A. The tumor pieces were then homogenized and centrifuged at 12,000 × g for 15 min at 4 °C. Solution C (solution A + 10% glycerol and 400 mM KCl) was added to the pellet in a 2:1 ratio (v/v) and vortexed on ice for 20 min. After centrifugation at 15,000*g* for 7 min, the resulting nuclear extract supernatant was collected in a chilled Eppendorf tube. Consensus oligonucleotides of NF-κB (Promega corporation, Madison, WI) was end-labeled using T4 polynucleotide kinase and (γ-32P) ATP for 10 min at 37 °C. Gel shift reactions were assembled and allowed to incubate at room temperature for 10 min followed by the addition of 1 μl (50,000 to 200,000 cpm) of ^32^P-labeled oligonucleotide and another 20 min of incubation at room temperature. Subsequently, 1 μl of gel loading buffer was added to each reaction and loaded onto a 4% nondenaturing gel and electrophoresed until the dye was 75% of the way down the gel. The gel was dried at 80 °C for 1 h and exposed to film overnight at 70 °C. The intensity of the bands was measured using the Fusion FX 7 image acquisition system (Vilber Lourmat, Eberhardzell, Germany).

### Chromatin Immunoprecipitation (ChIP) assay

ChIP analysis was performed using ChiP Assay Kit (Millipore) as described in the manufacturer’s instruction. Immunoprecipitated DNA was precipitated with ethanol and resuspended in 20 μl of ddH_2_O. PCR amplification of immunoprecipitated DNA was performed using the primers consisting of the oligonucleotides that encompass the promoter region of TIMP-3. The following primer sequences were used for TIMP-3 ChIP: the forward primer, 5′-GCGCCGGAGGCCAAGGTTGC-3′ and the reverse primer, 5′-CAGTCCCCCAGGCTCCAGCTGC-3′. The PCR products were separated on 1% agarose gels and analyzed using ethidium bromide staining. All ChIP assays were performed at least twice with similar results.

### Data analysis

The data were analyzed using the GraphPad Prism 4 ver. 4.03 software (GraphPad Software, La Jolla, CA). Data are presented as mean ± SD. The differences in all data were assessed by one-way analysis of variance (ANOVA). When the *P* value in the ANOVA test indicated statistical significance, the differences were assessed by the Dunnett’s test. A value of *P* < 0.05 was considered to be statistically significant.

## Electronic supplementary material

Supplementary Figure Legends

Supplementary Figures
