# Supplementary material for: IL-32 gamma reduces lung tumor development through upregulation of TIMP-3 overexpression and hypomethylation
Source: Cell Death Dis. 2018 Feb 21;9(3):306. doi: 10.1038/s41419-018-0375-6 (PMC5833366; doi:10.1038/s41419-018-0375-6)
Supplement: Supplementary file 1 — Supplementary Figure Legends [file 41419_2018_375_MOESM1_ESM.docx]

**Supplementary Figure Legends**

**Supplementary Fig. 1. A,** Expression pattern of TIMP3 and IL-32 in several organisms of IL-32γ Tg mice determined by Western blotting. Each band is representative for three experiments. **B**, Cytokines level was measured in the tumor tissues (three tissues/group)

**Supplementary Fig. 2.** Effect of IL-32γ on the promoter methylation of TIMP-3 in other cancer cell lines. Cancer cells were transfected with pcDNA or IL-32γ plasmid for 24 hr. TIMP-3 promoter methylation levels were determined by quantitative methylation-specific PCR by qPCR (**A**) and PCR (**B**) by using the specified primers.

**Supplementary Fig. 3.** **A**, Effect of other types of IL-32 on the promoter methylation of TIMP-3. Lung cancer cells were transfected with IL-32α or L-32β for 24 hr. TIMP-3 promoter methylation levels were determined by quantitative methylation-specific PCR 3 methylation by qPCR. **B,** Effect of IL-32γ on other genes methylation. Lung cancer cells were transfected with IL-32γ for 24 hr, and promoter methylation of other genes including E-cadherin, CDO1, TERT and p16 was determined by quantitative methylation-specific PCR 3 methylation by qPCR. **C**. Effect of Effect of siRNA IL-32γ on TIMP3 methylation and and expression. Lung cancer cell (A549) was transfected with IL-32γ for 24 hr, and then 24 hr after the cells were transfected again with siRNA. TIMP3 methylation was determined by quantitative methylation-specific PCR 3, and the expression was determined by Western blotting. Each band is representative for three experiments.
