# Supplementary material for: IL-32 gamma reduces lung tumor development through upregulation of TIMP-3 overexpression and hypomethylation
Source: Cell Death Dis. 2018 Feb 21;9(3):306. doi: 10.1038/s41419-018-0375-6 (PMC5833366; doi:10.1038/s41419-018-0375-6)
Supplement: Supplementary file 2 — Supplementary Figures [file 41419_2018_375_MOESM2_ESM.pptx]

## Slide 1
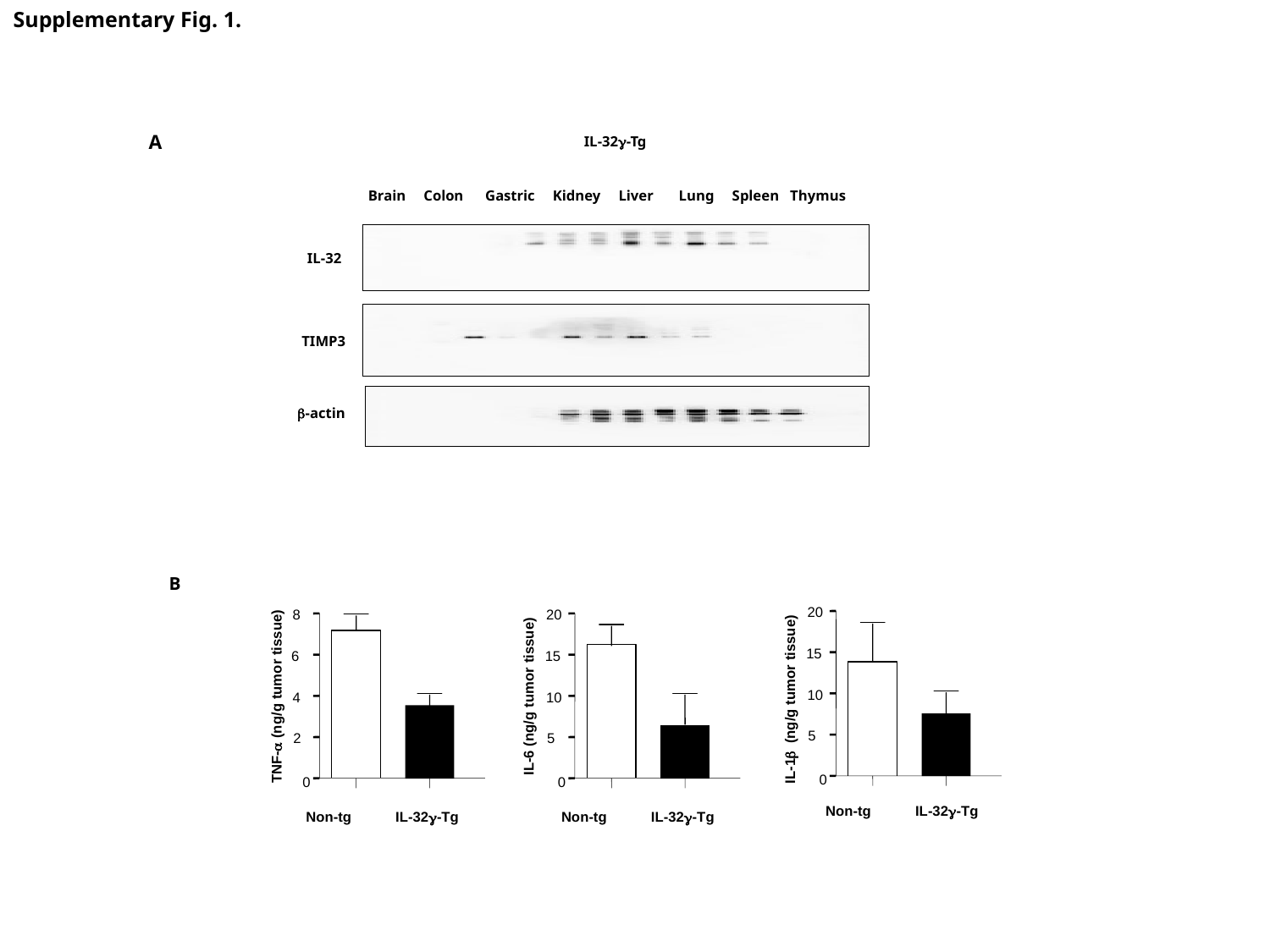

Supplementary Fig. 1.
A
IL-32g-Tg
Brain Colon Gastric Kidney Liver Lung Spleen Thymus
IL-32
TIMP3
b-actin
20
15
10
5
0
8
6
4
2
0
20
15
10
5
0
TNF-a (ng/g tumor tissue)
IL-1b (ng/g tumor tissue)
Non-tg IL-32g-Tg
Non-tg IL-32g-Tg
Non-tg IL-32g-Tg
B
IL-6 (ng/g tumor tissue)

## Slide 2
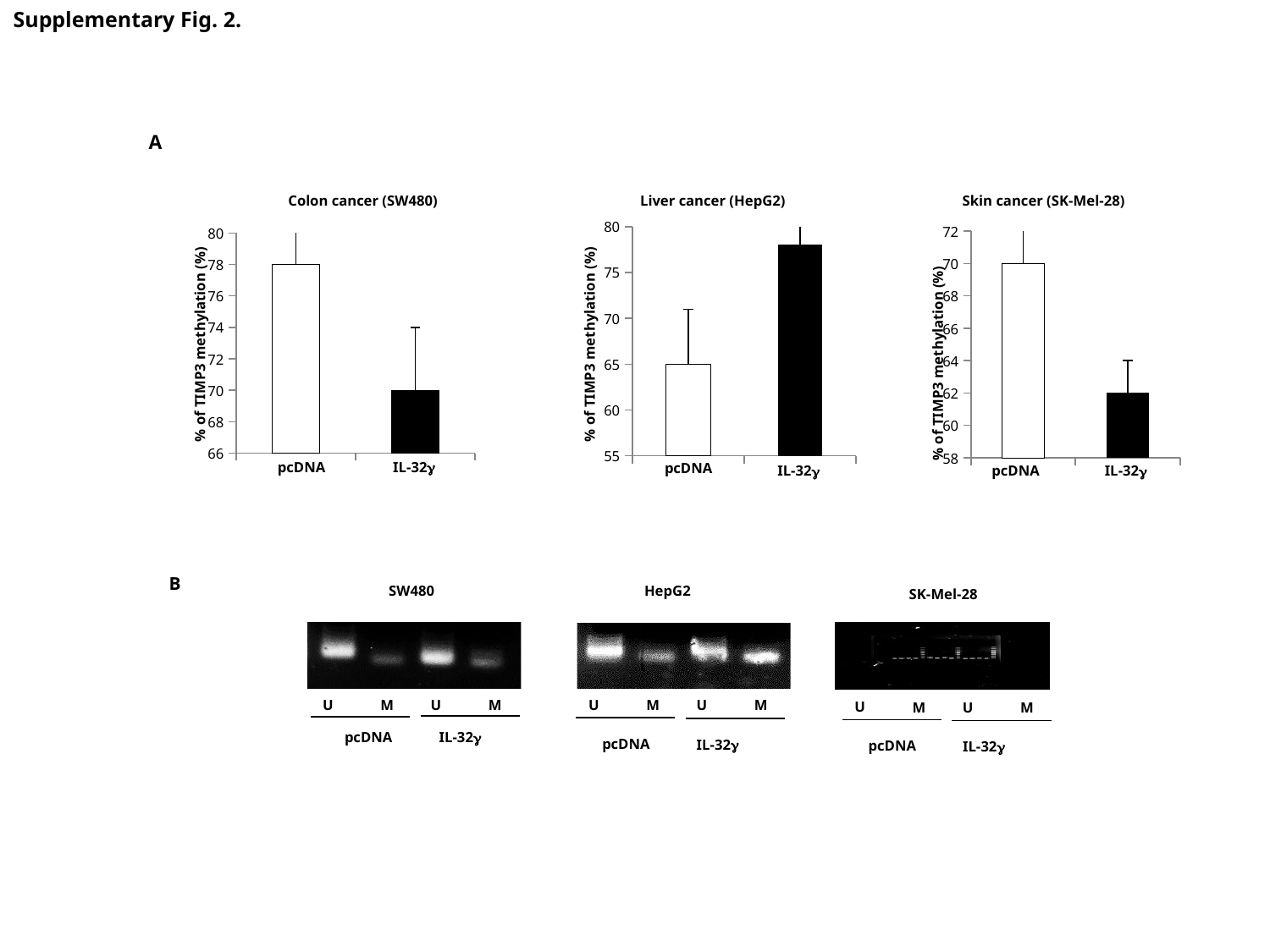

Supplementary Fig. 2.
A
Colon cancer (SW480)
Liver cancer (HepG2)
Skin cancer (SK-Mel-28)
### Chart
| Category | |
|---|---|
### Chart
| Category | |
|---|---|
### Chart
| Category | |
|---|---|% of TIMP3 methylation (%)
% of TIMP3 methylation (%)
% of TIMP3 methylation (%)
pcDNA
IL-32g
pcDNA
pcDNA
IL-32g
IL-32g
B
SW480
HepG2
SK-Mel-28
U
U
M
U
M
U
M
M
U
M
U
M
pcDNA
IL-32g
pcDNA
IL-32g
pcDNA
IL-32g

## Slide 3
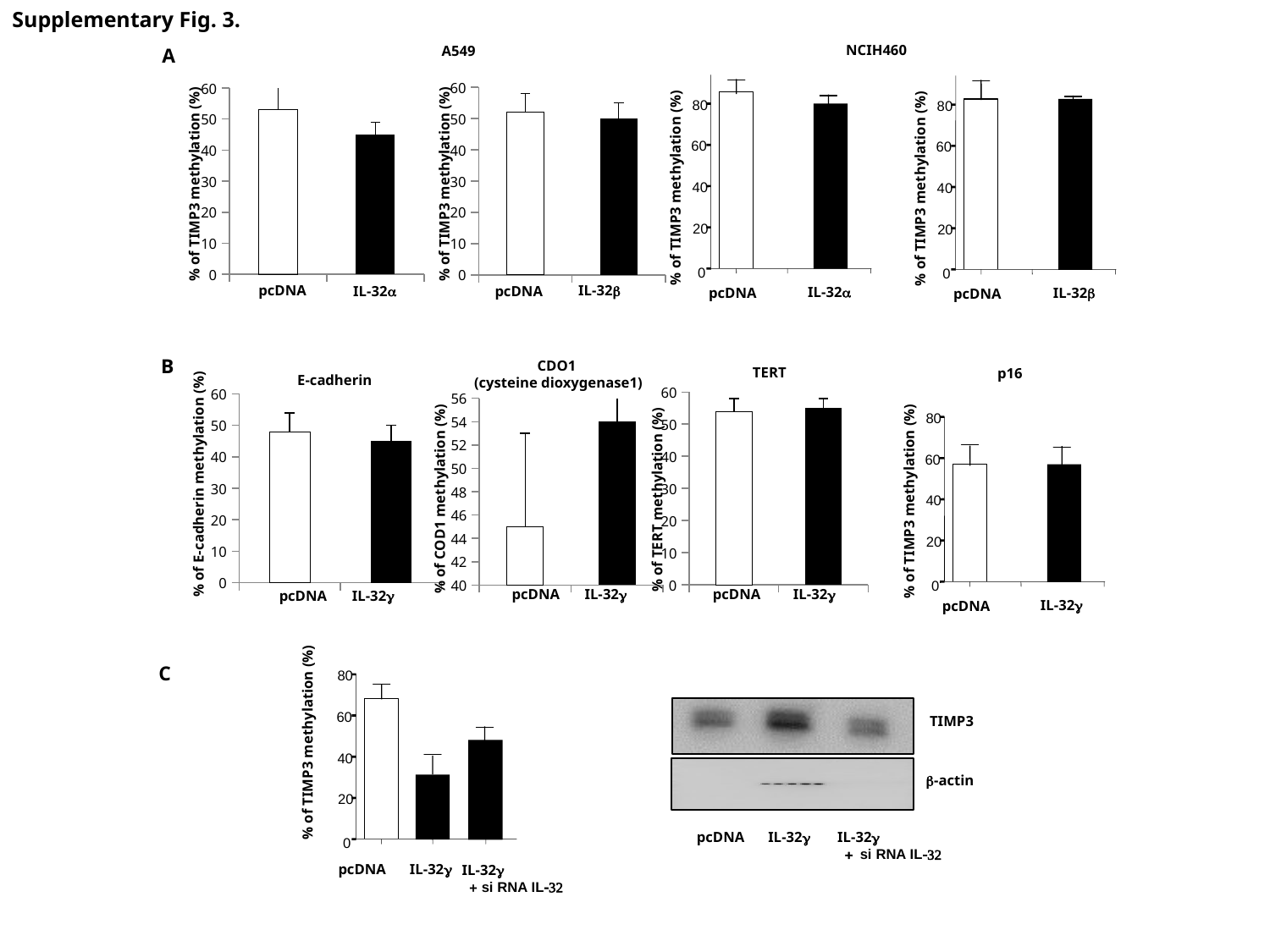

Supplementary Fig. 3.
NCIH460
A549
A
### Chart
| Category | |
|---|---|
### Chart
| Category | |
|---|---|
80
60
40
20
0
80
60
40
20
0
% of TIMP3 methylation (%)
% of TIMP3 methylation (%)
% of TIMP3 methylation (%)
% of TIMP3 methylation (%)
pcDNA
IL-32b
IL-32a
pcDNA
IL-32a
pcDNA
IL-32b
pcDNA
B
CDO1
(cysteine dioxygenase1)
TERT
p16
E-cadherin
### Chart
| Category | |
|---|---|
### Chart
| Category | |
|---|---|
### Chart
| Category | |
|---|---|80
60
40
20
0
% of E-cadherin methylation (%)
% of TERT methylation (%)
% of TIMP3 methylation (%)
% of COD1 methylation (%)
pcDNA
pcDNA
IL-32g
IL-32g
pcDNA
IL-32g
IL-32g
pcDNA
80
60
40
20
0
TIMP3
% of TIMP3 methylation (%)
b-actin
pcDNA
IL-32g
 + si RNA IL-32
IL-32g
pcDNA
IL-32g
IL-32g
 + si RNA IL-32
C
